# Concurrence of Congenital Muscular Torticollis and Congenital Torticollis Due to Other Anomalies: Two Case Reports

**DOI:** 10.3389/fped.2021.709616

**Published:** 2021-10-27

**Authors:** Min-Wook Kim, Da-Ye Kim, Dong-Woo Lee, Da-Hye Ryoo, Jaewon Kim, Dae-Hyun Jang

**Affiliations:** Department of Rehabilitation Medicine, College of Medicine, Incheon St Mary's Hospital, The Catholic University of Korea, Seoul, South Korea

**Keywords:** congenital torticollis, fibroma, trochlear nerve, ocular motility disorders, oculomotor muscles, vertebral anomaly

## Abstract

**Introduction:** Congenital muscular torticollis (CMT) is the most common cause of torticollis in infants; other causes, including osseous, ocular, and central nervous system torticollis can easily be overlooked. We report two rare cases of CMT with concurrent osseous or ocular torticollis.

**Case 1:** A 1-month-old female infant with a right neck mass and right-tilting head posture was referred. Neck ultrasonography showed diffuse hypertrophy and hyperechoic findings on the right sternocleidomastoid (SCM) muscle, which was consistent with right CMT. A clavicle X-ray imaging was conducted to identify an associated fracture due to birth trauma on the same day and a suspected congenital vertebral anomaly was coincidentally found. Subsequent three-dimensional computed tomography of the cervical spine showed a T1 hemivertebra causing the right-tilting head. The patient was diagnosed with the concurrent manifestation of CMT and congenital osseous torticollis.

**Case 2:** A 3-month-old male infant with a 20° head tilt to the right with a limited cervical range of motion was referred. Neck ultrasonography showed a fibromatosis colli in the right SCM, suggesting CMT. He proceeded to physical therapy for seven months; however, there was little clinical improvement in his head and neck posture. The patient underwent an additional ophthalmologic examination and orbital magnetic resonance imaging (MRI) at 10 months of age. The result showed congenital agenesis of the left fourth cranial nerve with hypoplasia of the superior oblique muscle causing the right-tilting of the head. Ultimately, the boy was diagnosed with a concurrent manifestation of CMT and congenital ocular torticollis.

**Conclusion:** Unless careful examinations are conducted, congenital vertebral anomalies and congenital agenesis of the fourth cranial nerve can go unnoticed in the present two cases. If a patient with CMT displays unusual features or does not respond to physical therapy, clinicians should consider not only a differential diagnosis but also concurrence with other causes of congenital torticollis.

## Introduction

Congenital torticollis, which presents as an abnormal posture of the head and neck, is a problem frequently encountered in pediatric rehabilitation clinics. Congenital muscular torticollis (CMT) due to shortening of the unilateral sternocleidomastoid (SCM) is the most common cause of congenital torticollis. Other causes include osseous, ocular, central/peripheral nervous system, and soft tissue torticollis (1–3). Early and accurate diagnosis, as well as proper management, are important because untreated or improperly treated patients with congenital torticollis are more likely to develop secondary conditions, such as plagiocephaly, scoliosis, and balance problems (4). CMT can be diagnosed with physical examinations such as observation of resting head and neck posture, palpation of SCM, and assessing of cervical active and passive range of motion (ROM) along with radiologic studies such as ultrasound examinations. CMT is usually categorized into 3 types: SCM mass, muscular (tightness of the SCM without an apparent mass), and postural torticollis with neither mass nor tightness (5–7). Whereas, causes of congenital torticollis other than CMT are likely to be overlooked because those are relatively uncommon and can be difficult to diagnose through physical examinations or plain X-rays in early infancy (8). Furthermore, if an infant is clinically confirmed through ultrasonography as having a CMT, other causes are typically ruled out. Two patients were recently seen at our institution with the initial diagnosis of CMT and co-existing conditions that showed as head tilt. The purpose of this report is to share the clinical pathway and necessary referrals that lead to the correct diagnosis and treatment of congenital torticollis.

## Case Description

### Case 1

**Figure 1:**
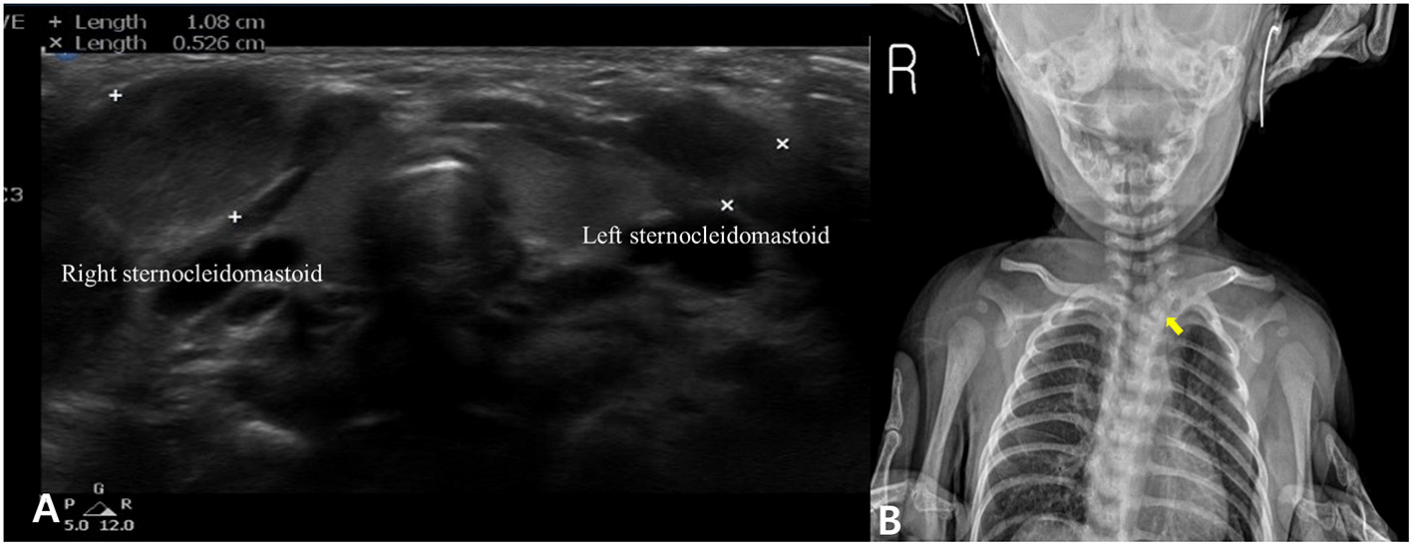
Ultrasonography showing diffuse enlargement and hyperechoic findings in the right sternocleidomastoid muscle **(A)**. A congenital vertebral anomaly was suspected in the clavicle X-ray imaging (yellow arrow) **(B)**.

**Figure 2:**
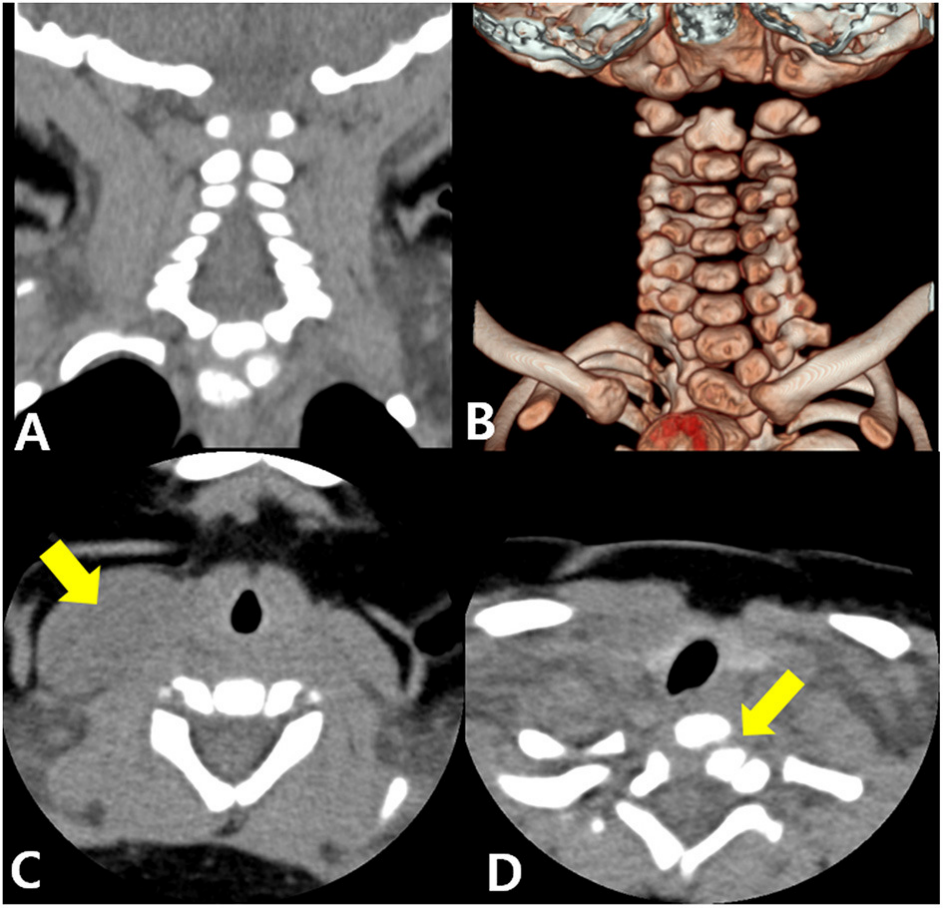
Three-dimensional volume-rendered computed tomography. Coronal image **(A)** and 3-dimensional image **(B)** showing T1 butterfly vertebra. Axial cervical image showing enlargement of the sternocleidomastoid muscle (arrow) at the C7 level **(C)** and a hemivertebra (arrow) at the T1 level **(D)**.

A 1-month-old female infant suspected of CMT was referred to our institution from a local clinic. A palpable mass in the right SCM and 10° head tilt to the right were noticed. Cervical passive ROM was measured by an arthrodial protractor described in a previous study (7). A 5° deficit in rotation to the right and a 10° deficit in lateral flexion to the left compared to the opposite side were revealed, respectively. The physiatrist, who specializes in musculoskeletal ultrasound medicine, used an X11 XE to perform an ultrasound examination of the SCM (Philips, Bothell, WA). Bilateral SCMs were studied from both a longitudinal and transverse perspective. Diffuse enlargement and hyperechoic findings on the right SCM were confirmed, which was compatible with the diagnosis of SCM mass type of CMT (Figure 1A). A clavicle X-ray imaging was conducted to identify an associated fracture due to birth trauma on the same day and a suspected congenital vertebral anomaly was coincidentally found (Figure 1B). Subsequent three-dimensional volume-rendered computed tomography (3D-CT) of the cervical spine revealed that the T1 hemivertebra caused the right tilt (Figures 2A–D). The patient was ultimately diagnosed with the concurrent manifestation of CMT and congenital osseous torticollis at 1-month-old age. As per the protocol of CMT management at our institution, a standardized program including manual stretching, strengthening exercises using postural reactions or visuo–auditory stimulations, and development of symmetric movement was administered two or three times weekly by a physiotherapist while attempting to avoid additional neurologic injury to the T1 hemivertebra for 12 months. The home exercise program was also implemented by educating caregivers (7, 8). Although the passive ROM had recovered completely, the head tilt to the right was still within ~5°. There were no neurological signs, and she showed normal development at the age of 24 months; therefore, she underwent no further treatment, with only her progress being monitored every 6 months so far (current age: 4 years old).

### Case 2

**Figure 3:**
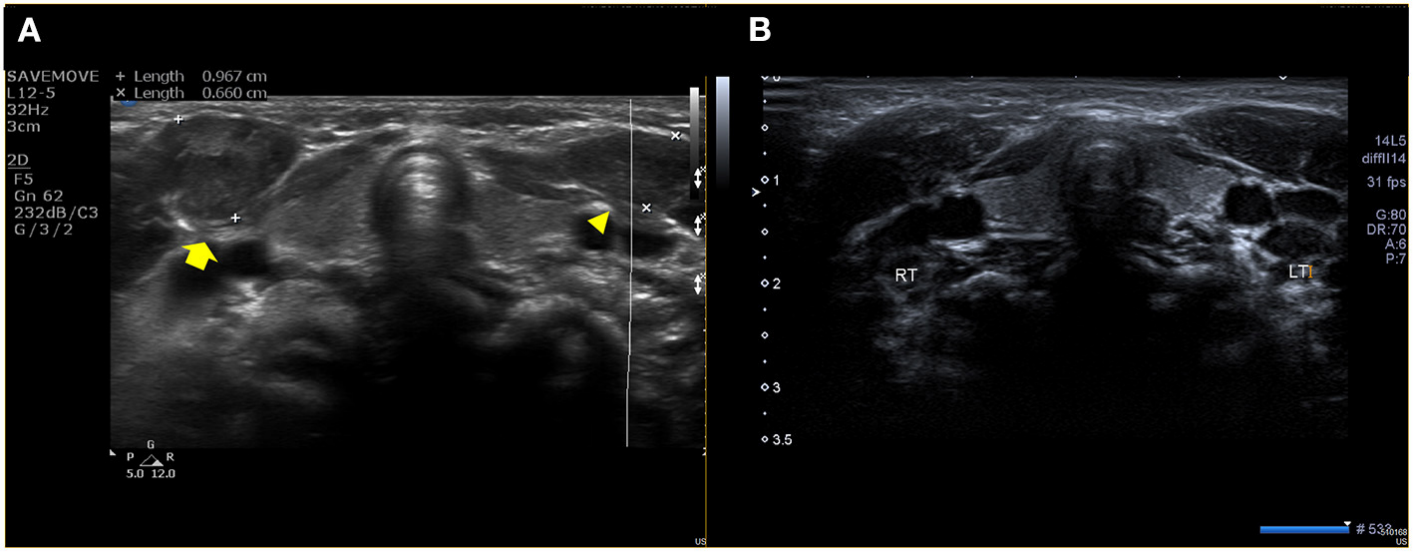
Ultrasonography of the right (yellow arrow) and left sternocleidomastoid muscles (arrowhead), revealing a fibromatosis colli in the right sternocleidomastoid, suggesting right CMT at the age of 3 months **(A)**. A follow-up test at the age of 7 months showed the decreased size and improved echogenicity in the right sternocleidomastoid **(B)**.

**Figure 4:**
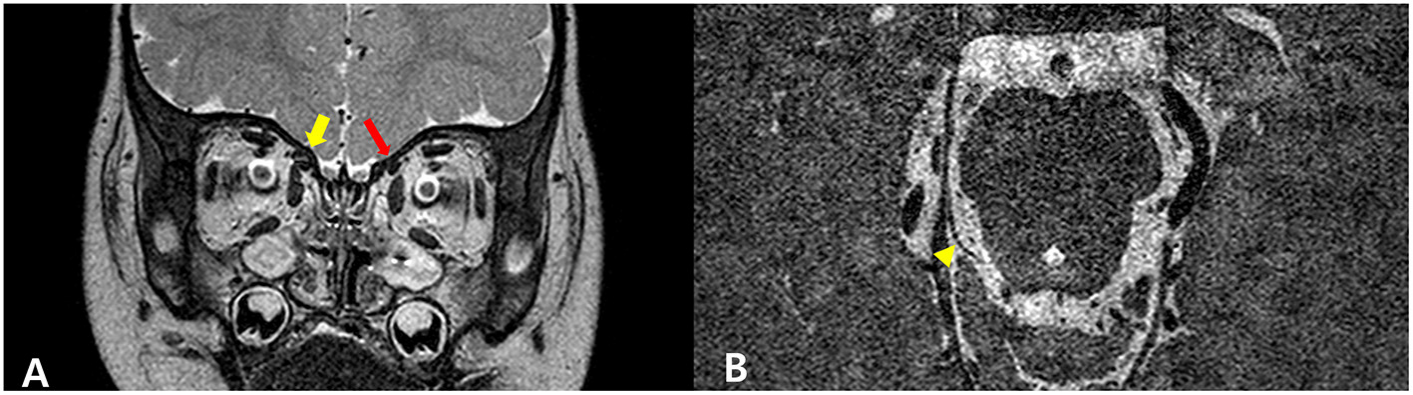
Hypoplasia of the left superior oblique muscle and an absence of the left fourth cranial nerve. Coronal T2-weighted orbital image showing that the left superior oblique muscle (red arrow) is smaller than the right (yellow arrow), suggesting hypoplasia **(A)**. A high-resolution image shows that the right fourth cranial nerve (arrow) is well-identified; however, the left fourth cranial nerve is not visible **(B)**.

A 3-month-old male infant was referred with suspected CMT. A clinical examination revealed a 20° head tilt to the right with a 45° deficit of cervical rotation ROM to the left compared to the right side. Plagiocephaly was evident on the left occiput; however, malposition of the affected ear and asymmetry of the forehead was slight. An ultrasound examination showed a fibromatosis colli and heterogeneous echogenicity in the right SCM, suggesting SCM mass type of CMT (Figure 3A). The patient underwent physical therapy for 3 months according to the CMT management protocol of our institution. Passive ROM of both cervical rotation and lateral flexion was improved completely; however, the 15° head tilt to the right was still evident. During follow-up, an ultrasound showed that the right SCM lesion had decreased in size and normalized echogenicity (Figure 3B). The patient continued physical therapy and home treatment. However, there was no additional improvement of the head tilt after four more months of treatment. There was no definite muscle tightness of the right SCM. Moreover, the symptoms were aggravated in the sitting position and when observing. The patient, therefore, underwent an additional ophthalmologic examination and orbit magnetic resonance imaging (MRI) at the age of 10 months. The examination revealed left hypertropia causing the right tilt, and the MRI revealed a small left superior oblique muscle and absence of the left fourth cranial nerve in the cistern (Figures 4A,B). The patient was ultimately diagnosed with the concurrent manifestation of CMT and congenital ocular torticollis. He underwent a left inferior oblique myectomy at the age of 15 months, and the torticollis was completely resolved. He had no additional physical therapy and was last monitored at 24-month-old age. The follow-up monitoring was ended because there was no head tilt or cervical ROM limitation.

## Discussion

Congenital torticollis is characterized by a clinical sign of a head tilt with rotation to the opposite side due to various pathogenic processes. It is caused by muscular, ocular, osseous, central/peripheral nervous system and soft tissue torticollis, etc. However, non-muscular causes can be easily overlooked because CMT accounts for most cases of torticollis in infants (3, 8, 9). For this reason, several studies have emphasized the importance of differentiating non-muscular causes while evaluating patients with torticollis (8, 10, 11).

Infants with abnormal head and neck posture should be carefully assessed to identify the exact cause. Clinical pathway guidelines suggest that the initial evaluation should include a description of resting posture, measurements of resting head tilt, cervical ROM into the rotation and lateral flexion, palpation of the SCM muscle for the possible mass, neurologic examination, and ophthalmologic examination (6, 8, 12). In addition to physical examinations, SCM muscle ultrasonography can be considered as the evaluation tool for CMT because of its high diagnostic validity (7). Other causes are clinically ruled out if the ultrasound reveals abnormalities in the unilateral SCM muscle, such as increased thickness, heterogeneous echogenicity, and a mass consistent with the diagnosis of CMT. In the present report, however, the two cases of congenital torticollis suggest the possibility of the concurrence of more than two underlying causes.

The first case revealed both CMT and congenital osseous torticollis of a vertebral anomaly. A recent study showed that ~1% of cases of congenital torticollis were caused by vertebral malformations. In addition, it suggested the importance of careful examinations and 3D-CT of the cervical spine because patients with congenital osseous torticollis might only show torticollis symptoms and ambiguous vertebral abnormalities on the cervical X-ray imaging just as our first patient showed a subtle abnormality on the clavicle X-ray imaging (8). Thus, clinicians should perform additional imaging tests to avoid misdiagnoses if the patient does not respond to physical therapy or normal cervical alignment and morphology are not evident on cervical X-rays.

The second case demonstrated the concurrence of CMT and congenital ocular torticollis due to agenesis of the fourth cranial nerve. Congenital ocular torticollis includes various etiologies, such as superior oblique muscle palsy (the most common), other ocular deviations, nystagmus, Brown syndrome, and spasmus nutans (3, 13, 14). The prevalence of congenital ocular torticollis is unclear but is considered more frequent than congenital osseous torticollis (8). Congenital ocular torticollis is diagnosed by employing several clinical tests, such as visual function, pupillary reactivity, and extraocular movement (duction/version) assessments, the corneal light reflex test, the cover/uncover test, and neuroimaging studies (15). The diagnosis of congenital ocular torticollis is typically more delayed than CMT because binocular vision development begins at ~4 months of age. Therefore, the condition is not suspected until the patients can sit independently and observe while in a sitting position (16). Our patient was diagnosed with CMT at the age of 3 months, and there was some improvement after the initial physical therapy. Despite continued therapy, he did not improve further. We, therefore, performed an ophthalmologic evaluation. Early diagnosis of congenital ocular torticollis is important because delayed management may lead to loss of binocularity, development of amblyopia, and permanent deformities caused by musculoskeletal system tissue changes (17). If a child's torticollis worsens while observing an object in a seated position or if the child is resistant to physiotherapy, ophthalmologic evaluations should be conducted (18).

Besides osseous and ocular causes, there are various underlying etiologies of torticollis such as infection, inflammation, tumor of the brain, spinal cord or cervical spine, and neurologic disorders in children. Therefore, healthcare providers should carefully implement initial and ongoing evaluations while managing CMT, particularly when the patient has atypical signs and symptoms which suggest non-muscular congenital torticollis. Red flags for further evaluation include atypical cranial deformation, acute-onset, late-onset at 6 months or older, abnormal neurologic signs, visual abnormalities, and worsening of or unresponsiveness to physical therapy (6, 19).

Our two patients presented with a coincidence of two causes of congenital torticollis in the same direction, thereby exacerbating the symptoms. Unless careful ongoing examination and assessment are conducted, congenital vertebral anomalies and congenital agenesis of the fourth cranial nerve might go unnoticed in such cases. If the concurrent types of congenital torticollis in such patients are not identified in their early state, surgical resection of the SCM muscle can be conducted in cases refractory to physical therapy. Clinicians should therefore consider not only the differential diagnosis but also the concurrence with other causes of congenital torticollis if patients with CMT present with unusual features or do not respond to conventional therapy.

## Data Availability Statement

The original contributions presented in the study are included in the article/supplementary material, further inquiries can be directed to the corresponding author/s.

## Author Contributions

M-WK: acquisition of data, analysis and interpretation of data, writing, and critical revision of the manuscript. D-YK, D-HR, JK, and D-WL: acquisition of data and analysis and interpretation of data. D-HJ: study concept and design, acquisition of data, analysis and interpretation of data, study supervision, and critical revision of the manuscript for intellectual content. All authors contributed to the article and approved the submitted version.

## Conflict of Interest

The authors declare that the research was conducted in the absence of any commercial or financial relationships that could be construed as a potential conflict of interest.

## Publisher's Note

All claims expressed in this article are solely those of the authors and do not necessarily represent those of their affiliated organizations, or those of the publisher, the editors and the reviewers. Any product that may be evaluated in this article, or claim that may be made by its manufacturer, is not guaranteed or endorsed by the publisher.

## Abbreviations

CMT: Congenital muscular torticollis
SCM: Sternocleidomastoid
MRI: Magnetic resonance imaging
ROM: Range of motion.
